# Sanitalogy: A Discussion of the Health Principle

**Published:** 1903-10

**Authors:** D. R. Stubblefield

**Affiliations:** Nashville, Tenn.


### SANITALOGY: A DISCUSSION OF THE HEALTH PRINCIPLE.[1]

BY D. R. STUBBLEFIELD, M.D., D.D.S., NASHVILLE, TENN.

THE world is not so large but what the feet of the traveller have trodden its farthest bounds. If one reason has failed to induce the investigation of any part of the globe, another has arisen to supply the demand. He is a rare traveller, then, who can bring back a story of new regions explored, new paths walked in, new scenes beheld. Of late years it has grown somewhat similar in the world of science. So much has been rendered commonplace by familiarity that even marvels would scarcely be deemed worthy of head-lines in the evening papers. It is small wonder we have doubted what would entertain you on this occasion.

While anxiously meditating, many of the trite old subjects that have done duty from the days of the fathers presented themselves. That long line of service-worn cripples did not look like a review of veterans, broken by the scars of honorable strife, and therefore entitled to sympathetic consideration, but seemed rather like an army of impudent beggars who showed that each one thought that he was entitled to "all that was coming." Just like beggars, the rebuff of one did not affect the confident air of the next, for each one had to have his own dose of direct dismissal before the preposterous idea of his rejection could gain lodgement in his cranium. For hours this was kept up. We were not willing to attempt to rehabilitate such scarecrows as most of them were, and we wearily turned the glass of our hope out into the seeming void of the unknown. Like another Barnard, we glued our eyes to the reflector and would not budge, held there by the belief that another "fifth satellite of Jupiter" was there if we could only pierce the tremulous depths of ether beyond. Failing in this, as a last resort we came home and began to take stock of all those fugitive ideas and half-formed convictions that had accumulated within us as the years had passed.

At last we found a trail of an idea, but could not find a tree to run it up. Think of that, an idea and no word to express it!

---

[1] Read before the Massachusetts Dental Society, Boston, June 3 and 4, 1903.

We were therefore compelled to coin a word, which, strictly interpreted, is open to the same objection as the synonyms in not exactly expressing our meaning; still, as it has no recognition by any one for anything else, it ought to be permitted to us herein. Our friend Dr. Boardman made the reassuring comment on it that we would keep everybody "guessing" with such a title. Nevertheless, when we are through we venture the prediction that none will be in doubt as to our word or meaning.

When we confess that the subject of this paper is an unusual though not unique union of two languages, a word from two very obvious roots, we are sure it will take the wind out of the sails of your criticism. As we have interpreted sanitalogy it is not sanitation, nor exactly hygiene, although suggesting both from an etymological stand-point. For the purpose of this discussion, whether it is exactly true or not, we are going to limit sanitation to those offices done by the State or city to improve environment and overcome those material menaces to public health which naturally and rapidly accumulate. This we shall call State medicine, comprising all efforts to remove or render innocuous the myriad forms of by-products, excretory in their nature, which result from great aggregations of human beings. It embraces all those acts that are done on the outside of us, around us, which the State or municipality not only may, but must, do as a matter of self-preservation. Very properly in this realm we place sewerage, street-keeping, garbage removal and disposal, drainage, general ventilation and disinfection, food examination, and so forth. We repeat that we are very well aware that all of this general health-precaution cannot be legitimately thus classified, but we make bold to do so for our purpose on this occasion.

Again, whether it is exactly true or not, we are going to confine hygiene, or hygeology, to all those intelligent services which the physician renders to his patient. He takes it for granted that his patient has received the benefits of all that sanitation can do, and in addition prescribes certain regimen or measures to further protect him from disease. This comes, you will please observe, closer to the individual, follows him into his home, in fact, and may be the just personal parallel to those measures included in State medicine, which we have applied to the community at large. It is a fact that at several points the two merge and we find that the State or city health officer joins the family physician in isolating contagious cases and complying with certain demands of a more general nature and extent. The family physician concentrates his powers on the individual and, forsaking all others for the time being, devotes himself to the needs of that one. He treats this organism in like manner that the ideal sanitary officer is expected to treat and guard the city or State at large. The sanitary officer uses quarantine, the cart, the hose, and, if deemed necessary, the removal or even destruction of an offending centre of disease; the hygienic officer uses the bath, the purge, the thousand and one combinations and concoctions upon which science or tradition, or both, depend, and if all these fail he sends his patient to Europe to rid himself of further embarrassment.

It will be noted that all these measures and endeavors proceed from the outside and go to the centre. The organism is the object aimed at by them. There is an honest intention that amply justifies all that is done, doubtless, but we desire to emphasize the fact that the co-operation of the individual is taken for granted or accepted as incidental and not always, if ever, recognized. *A power working from within outward* is the key, the motif, the declared entity of our subject and this discussion. It might be termed vitality, nature, or the "vis medicatrix naturæ," but we shall call it the health principle. It might be suggested that the study of biology would cover the subject, but that investigation of the cell, with all its processes, changes, and phenomena, is still outside the intention of this discussion. The cell is admitted into this discussion as the priordial organism in which the necessity for outside assistance and care is measured in parallel by that which is called for by the whole organism. It is a form of living structure that differs in degree but not in kind. It may be affected by the outside measures before cited, and it is, though infinitesimal, the theatre of action of that principle above denominated the health principle, as much so as if it were greatly enlarged. We do not mean to speak here of material structure as such, but only as the vehicle, organ, or home of this health principle within us. What shall be said regarding this pervading principle is presented as merely suggestive rather than any attempt at final conclusion or scientific contribution. Reflections upon the subject have for a long time entertained us, and of late years have greatly modified the views taken of many of the so-called dogmas which still dominate the world in every department of medicine and surgery.

That there is such a principle or power within all living organisms cannot be doubted. There is a tendency in all organic life, whether animal or vegetable, to defend itself and repair itself, to get well from any lesion or interference with its normal state and maintain that condition. It is the sheet-anchor of the confidence, conscious or unconscious, of every practitioner of the healing art, whether he treats a man or a monkey or a molecule of protoplasm. Like many other natural beneficences, however, it has been taken for granted and little valued, yet its indefinite suspension or loss would mean the rapid cessation of all organic life. Much has been done to protect the citizen from the evil effects of all causes of pathological conditions; much has been done by the physician to enable him to prove the efficient guardian of the health of those who depend upon him; but very little has been done with any definite idea of trying to reach that inner realm of life, where the battle of life is won or lost, to study its ruling spirit, to attempt to know its nature and properties that may be developed or at least husbanded. Very little has been found out about it, and what has been ascertained has not been classified as such.

We might say, first, that it certainly exists in every living organism as a special and peculiar part of that organism. It is not only there, but it is different in the individual of the class, and no rule has been ascertained by which its qualities may be certainly known. In the human species there are many individual personal differences, even where the anatomical similarities seem to indicate that there may be two identically alike somewhere. No one who has thought about it wonders that temperament, sex, race, environment, and other influences produce dissimiliarities in individuals that widely separate them. Even the recognized force of heredity is not proof against this individualizing power, whatever it is, and we find dissimilarities so marked between those closely allied by blood that they are incongruous. So much is this true that while the power of probability is quite confidently relied upon, still we must in the end recognize that breeding, like chemistry, is an experimental science, the results of which you may never know until you have obtained a practical result; but, unlike chemistry, you cannot repeat any definite achievement. And, therefore, no one should wonder when we suggest that this elusive health principle, though resident in every one, is individual in each organism of the human family. We hear it said that size, everything being equal, gives strength, and yet we know that everything else is not equal, nor can be. The physician cannot tell where or in whom he can find a certain co-operative force, but he recognizes it when he does find it, and rejoices in its potent assistance. A large person is apt to assume that because his typal demand has carried him beyond his fellows in size he may look with scorn upon one whose littleness makes him sensitive to comparative references, but everybody knows of illustrations from life wherein the giant has failed in feats of endurance when the middle-sized man has held the same strain lightly upon his shoulders. Speculations abound regarding the part that the color of the eyes or hair, the smoothness or roughness of the skin, play in settling this question, and about the time that speculation concludes with certainty, a fact from real life knocks the conclusion all to pieces. A great coarse man, knotted with muscles, covered with curled hair like a bull, glories in the puny effect that privation has made upon him. That same man may survive the rest of the crew cast away at sea, but many instances have told the utter unreliability of such confidence. The learned doctors tell us that the system of the chronic invalid has become so inured to unsanitary conditions, partial suffocation, or like strain upon the constitution, that he will survive those who have not had the training of privations to fit them for the task; but about the time you accept this plausible solution to fit a certain case, you hear the profession say that a certain locality has had such a depressing effect upon its inhabitants that it is no wonder they died like sheep when the pestilence came. All the theories regarding the quality of this power in the individual have failed about the time they were substantiated, if not before. The best we can do is to admit that each has it and that its properties are proved by actual results, and then acknowledge that the results are about as lasting as the tracings on the sands by the sea. While we cannot prove it, we must believe that size and sex, age and action, afford a basis for present probabilities, but for certainty against all emergencies there are no infallible cognitions. We are forced to admit as true what all must accept, that in this as in all else each man is an individual with enough that is common in him to establish his kinship to the rest of the human family, but represents after all a most carefully differentiated individual by a thousand indisputable tests.

So much for the principle itself. It exists, and when strong it holds us up, and when weak it fails us in the time of need. Every man is a law unto himself with regard to its quality, and no analysis of laboratory, nor investigation of microscope, nor refinement of human judgment has ever been able to measure it unerringly. However, we are not entirely in the dark. There are some things we may know and confidently rely upon for all practical purposes. In the first place, this health principle will bear no codling without proportionate loss of power. Hard usage, if it is not too hard and too long maintained, seems to be its best treatment. When you are tired you lie down to rest, and nature is grateful for the opportunity to restore the tone of the associated parts called the system, but if you stay in bed long after the time necessary to recuperate you begin to make inroad upon the fibre of your physical constitution. How much this law of use and disuse enters into the realm occupied by this health principle is difficult to determine, but that it plays a part cannot well be doubted. It must be remembered that the two sides are equally important. It is just as necessary to use enough as it is to not disuse beyond the proper point, and the nicest judgment is required to steer unerringly between the two in every case. Strange to say, in the wisest application of action, each would better be trusted to direct for another than for himself. Therein is found the most powerful reason for the existence of the family physician, because, apart from any benefit he may confer by reason of his scientific attainments, he views the organism, knowing its special weaknesses by frequent and interested observation, from another and outside viewpoint from the man himself, and is therefore better able to advise.

Not only must overkindness be avoided to foster this health principle, but it is known that getting close to nature helps. This phrase, literally interpreted, means to get out into the sunshine, where the air is nearest to its ideal composition,—oxygen one and nitrogen four parts,—and then do something to promote activity among the vital processes. The trouble about most people is that because certain functions were set in motion before intelligent perception was enthroned, they never seem to realize that they either should or can take any part in their special performance. Ask any pulmonary specialist how many human lungs he ever examined whose every air-vesicle was habitually or even occasionally brought into highest functional activity, and hear him reply: "None or next to none." Therefore, when you have gone out into the pure air, where you will necessarily use an improved quality of air in your lungs, you have not done your whole duty until you have caused by intelligent direction every single air-cell to be stretched by many full inspirations. And if this is true when you have the very best quality of air, what reason can there be for not doing the same all the time with the best you have? Indeed, is not the demand more imperative when you have an inferior quality? Remember that there is no atmosphere, however much vitiated it seems with matters that offend the eye or nose, that is without the health-giving oxygen, which endosmosis will separate and load on to those willing carriers, the red corpuscles, and they in turn will feed the hungry molecules within us if they have half a chance. If mankind were well and happy physically only when supplied with ideally pure air, then this race would be born only to die again. If the filter-plant of osmosis were able only to handle pure air in the lungs, if the average individual could not withstand the relatively small imposition of the ordinary city air, then it would not take a learned mathematician to compute the number of breaths in the world.

In addition to these reflections upon air and breathing as adjuvants and fostering influences upon our health principle, why might we not as wisely speculate upon the use of sunshine? We all know what bleaches the felon in the murky jail and causes the pallid features of the bedridden invalid, but we do not associate the two results as identical except upon plain reasoning. We say confinement causes the one and sickness produces the other, but we fail to fully realize that while different causes are at work similar consequences are coming to view, and we know that while sunshine may not be able to alone cure the sickness, it will certainly relieve the pallor. Therefore, it is the part of wisdom to not only go into the sunshine, but to put yourself in proper relation to its health-giving power. Most of us look upon the sunbath as the fad of a quack, as it is ordinarily presented to our minds, but no man who thinks with the mind of a doctor can doubt its efficacy. In fact, no one knows the limit of power of these natural agencies when brought to bear upon the organism suffering from the baleful influences that form so great a part of our false living. The wonder is not that so few have perfect health, but that any at all come anywhere near the proper standard of normal function, beset as we are from our cradles with ignorance and sloth and all the devitalizing influences arising from both. How many infants are allowed to enjoy the naturally normal tone of the newly born? The thousand and one interferences with proper vital function that are forced upon these little strangers have engendered a superstitious belief that babes are born reeking with all manner of infantile perversions. The average nurse (much more the average young parent) believes every child is born ravenously hungry, and interprets his involuntary commencement of his physical development into a series of frenzied efforts to rake in the whole world and straightway swallow it. How long do they have the privilege of being let alone at least? Who ever heard of even a learned doctor who would patiently wait for time to develop hunger in an infant, as he knows is necessary in every other organism? No; the usual plan is to go to work upon the first squall to force down his innocent throat, if not some vile decoction, certainly an aliment that he cannot digest any better than he can digest a stone. Anybody would stand aghast if you suggested to put his little mind studying logarithms or the intricacies of logic, but no one seems to think that his digestive apparatus is, in parallel, just as rudimentary as his brain development. Colic? Of course, he has colic. Weak as he is,—because he is such a little bud of humanity,—the health principle in him makes a strenuous effort to rid him of those vile offenders which have been ruthlessly forced down him. There are grown people who unaccountably believe that anything that can be swallowed, even if it takes several attempts to accomplish it, will be in some unreasoning way digested. How often did you ever hear of mothers—God bless them—poisoning their own infants by chewing up some food fit only for their own adult digestion, and by persistent efforts getting it down those little throats? And then they walk the floor (for they cannot get others who shall be nameless here to do it for them), upheld through the long nights by a devotion worthier to be sung than any endurance exhibited on a Polar expedition. But, oh, the pity of it. If the health principle were not strong within us, if we could not withstand impositions from the first hour through our lives, none of us would ever live to vote, much less live to be President of these United States.

And then there is another natural remedy that is not appreciated at anything like its true value. It is water. Water, the greatest component of proud man,—and we might say peerless woman, also,—is not only priceless in value, but valueless in price to most of us, from the high estimate that should be put upon it. It should be used like the old fellow said whiskey should be to get the proper benefit from it, "Externally, internally, and eternally." It is Nature's great solvent. We very naturally turn to it to remove almost all the defilements of life. But for its value outside, its greatest value and highest worth comes from its marvellous utility inside the human organism. It is the great menstruum and diluent of all the fluids of the human body. It is the great cleanser of every excretory gland, the great flusher of the myriad sewer channels of our personal municipality. Its great importance may be fairly inferred when we compare the desiccated body—a mere handful—with the weight taken in full health. If the Creator found it necessary to utilize so much water in our composition, why do we cultivate almost a hydrophobia all our lives? Again, we are reminded that the use of water to any large extent as a remedial agent is popularly relegated to a special kind of doctor, and a plea for its more generous, more intelligent use will be taken as a suggestion for hydropathy. Not so. But we believe that we are regularly making specimens of arrested development of our children and needlessly hindering a proper action of our own organs by our senseless disregard of one of Nature's best gifts to man. We habituate ourselves to do without water until we actually believe it is best to live on half-rations all our lives. Again we say if we could not stand up under countless and constant impositions we would soon put on immortality. Every now and then some crank—a man who does his own thinking—gets upon a house-top and shouts aloud the marvellous benefits he has received from the intelligent use of one or all of these natural blessings, and we shake our wise heads or tap them significantly with a nod towards the speaker. Should we need argument to convince us of their usefulness? It would be enough to put any of us on the witness-stand and let the result depend on our own responses. Every bacteriologist knows that moisture is just as much a vital necessity to any germ—the black devil of the present age—as it is to have heat or food to sustain its life. Then why is it unreasonable or illogical for us to declare that its abundant use —following after nature—would be the highest wisdom?

Let us devote a few moments to another phase,—that of the use of what might be called the unnatural remedies in their effect upon this health principle. In the layman's mind there is absolutely no doubt of the power of drugs to produce a cure. To cure a disease by means of drugs seems to him no more wonderful than to patch up a piece of china with a little cement. We wonder if such a frame of mind is altogether peculiar to the layman? Does any one here happen to know of a doctor who seems to believe in the infallibility of his own prescriptions upon all—save himself? There is a time-honored saw that says, "No doctor takes his own medicine." If there ever was enough of basal truth to give that maxim life enough to live, why has it not proved sufficient to raise a broader question in the minds of the best of the profession? Who can say that there does not exist in the minds of the best doctors to-day much strong conviction that drugs of themselves are not to be depended upon? And this attitude of mind is not new. Your own Dr. Oliver Wendell Holmes is recorded as saying, "Nature cures; Art helps if she can, but oftener hinders." Upon the last analysis is it not true that drugs must forever be foreign matters to our economy? Are they not irritants, as it were, producing reactionary efforts on the part of this very power of which we have been speaking? These efforts may result beneficially, and for that reason we may not decry them, but as to their intrinsic nature and effect are they not in a sense the lesser evil, at the highest valuation? A spur to a horse may quicken his vitality, but the horseman who wishes to go a long journey knows he must beware the early and inconsiderate use of the spur. Indeed, to declare the constant use of the whip as necessary would not be considered good horse-trading sense. The "fear of punishment or the hope of reward" might be discussed in other places than our country schools. We cannot but believe the punishment produced by the more or less incongruous drugs may be at times beneficial, but, as a rule, the parts of the system have their work cut out for them to recover from their demoralizing effects. We do not wish to be thought as setting ourselves up against the intelligent physicians or as declaring war on the doctors without discrimination, but we want to ask of each practitioner how many so-called doctors he knows that he would not trust himself in the hands of unquestioningly? And the fundamental reason for his lack of faith in the individual is due to his unconscious admission that there is little if any faith to be put in a mere drug that is capable of harm. We are well aware how delicate this topic is, how difficult it would be to defend one's self in court against the almost unanimous indignation that might blaze up, but we mean to settle this suit before the tribunal of every honest thinker's consciousness. Let him who can deny its probability in the quiet chamber of his own soul, who can say that he unqualifiedly believes in the dogmas of the doctors, be the one to cast the first stone. Of course, the practice of medicine is valuable to the world, both because of the helplessness of the average inhabitant without it and because the intelligent physician earns his fee and more, as we have already said, by judicious advice and services, but after all it must be forever a system of playing upon the health principle within us. And therefore, so long as man is man, incapable of doing his own thinking, the race must struggle along from births to deaths, enduring the blunders at home and out of it and blindly expiating a thousand crimes against the heaven-given guardian presiding within every living organism.

One word in application to our own specialty, and we have finished. What has been said generally is true specially, although from the nature of the tissue involved it is not always so easily manifest. Long years ago our theory of dental caries was a chemico-vital breakdown of the teeth. We fancy we have gotten away from that definition in this enlightened age, because we have been able to find the germs and their acid by-products as sufficient causes for discerned results. But chemical action is still recognized, and the modification due to the presence of vitality, let us call it, is also admitted in the best observations of our laboratories. In miniature and in modified measure we can discern all the pathological changes in tooth-structure that are recognized in soft tissue. Response to the causal irritant is none the less certain because in hard tissue the cardinal symptoms of inflammation are necessarily modified by that structure. And the presence and power of the health principle in teeth may be as unquestionably shown in them as in other parts. Immunity from caries, so abundant in some, yet more or less present in all mouths, cannot be due to the absence of germs, for they are present in all oral cavities. Some teeth, we say, resist better than others, and let it go at that, but what enables them to resist? In the same line, why does the epidemic fail to affect all, or doctor and patient alike? What is vital resistance, if it is not the power that we have been discussing?

Again, when the teeth are involved every dentist knows that some unconditionally yield to harmful causes, fail to be benefited by the same reparative measures that are successful in others, and in many ways show a lack of a health principle. In others, in the same professional hands, the opposite is true. Yet, mind you, the germs—the causes of disease—are present in every mouth, and the human mouth is known to be the ideal incubator, with heat, moisture, and food always present. What is so-called secondary dentine but a provisional wall built against the irritating approach of disease or the presence of a foreign body put there as a safeguard against other intrusions? We see all the time the greatest anomaly of all surgery,—death tolerated more or less kindly by living tissue,—and yet we ask to be shown some open evidence of the practical presence of the health principle, so easily seen elsewhere. Dr. E. S. Talbot says that "auto-infection" is the cause of "interstitial gingivitis," and if there is a better explanation of pyorrhœa alveolaris, if you prefer that name, we are not aware of it. Self-poisoning is not only possible, but almost unavoidable, especially if we fail to use what nature and art have placed at our disposal, as most of us do continually. Verily, there's no health in us, despite our health principle, because that is true. But what suggestions are embodied in the best treatment of the disease referred to? Fresh air, generous diet, pure water, and the generally bracing and uplifting results of an ocean voyage are all adjuvants that all agree and rely upon to aid our best efforts otherwise. These are the very agencies which have been indicated as the best methods to sustain and develop and co-operate with the health principle. In conclusion, we affirm that whether we believe it or not, whether we can admit the tangibility or not, there is a health principle, and its intelligent study is the essence of that classic injunction, "Know thyself."

